# Female hormone therapy and risk of intracranial hemorrhage and focal neurological deficits in patients with cavernous malformations of the central nervous system

**DOI:** 10.3389/fneur.2025.1658980

**Published:** 2025-12-17

**Authors:** Saskia Wildi, Selina Nager, Victor Staartjes, Vittorio Stumpo, Sena Özkaratufan, Niklaus Krayenbühl, Oliver Bozinov, Luca Regli, Julia Velz

**Affiliations:** 1 Department of Neurosurgery, Clinical Neuroscience Center, University Hospital Zurich, Zurich, Switzerland; 2 University of Zurich, Zurich, Switzerland; 3 Division of Pediatric Neurosurgery, University Children’s Hospital Zurich, Zurich, Switzerland; 4 Department of Neurosurgery, HOCH Health Ostschweiz, Cantonsspital St. Gallen, St. Gallen, Switzerland

**Keywords:** female hormone therapy, intracranial hemorrhage, neurological deficit, cerebral cavernous malformation, cavernoma

## Abstract

**Background:**

Female hormone therapy [FHT, birth control treatment and postmenopausal hormone replacement therapy (HRT)] is not withheld from patients with cerebral cavernous malformations (CCM), notwithstanding the uncertainty surrounding the impact of these medications on the risk of intracranial hemorrhage (ICH). This study aimed to evaluate the impact of female hormone therapy on the risk of ICH or focal neurological deficit (FND) in patients with CCM.

**Methods:**

The prospective institutional database was examined for all patients with CCM treated at our institution between 2006 and 2023. Inclusion criteria comprised patients with confirmed CCM diagnosis through radiological and/or histological examination, availability of baseline clinical characteristics, accessible medication history, and follow-up data. Patients were stratified based on their medical treatment regimen, which included female hormone therapy or no treatment. The study assessed the time-to-event probability and the number of events (ICH or FND) during the follow-up period.

**Results:**

A total of 328 female patients with CCM were included in the final analysis. Among them, 56 patients (17.1%) were receiving female hormone therapy. Specifically, 37 patients (11.3%) were using birth control treatments and 19 patients (5.8%) were on HRT. The mean number of ICH per patient was 0.43 (SD 1.11) in the FHT group and 0.38 (SD 0.8) in the control group (*p* = 0.1), while the mean number of FND was 0.36 (SD 0.84) in the FHT group and 0.28 (SD 0.66) in the control group (*p* = 0.58). The time to first ICH was 1631.5 days (SD 2324.6) in the FHT group and 1161.4 days (SD 1650.8) in the control group (*p* = 0.35), while the time to first FND was 1601.0 days (SD 1934.1) in the FHT group and 1208.1 days (SD 1649.5) in the control group (*p* = 0.86).

**Conclusion:**

The study shows that female patients with a diagnosis of cerebral cavernous malformation receiving female hormone therapy do not experience a significant higher likelihood of intracranial hemorrhage or focal neurological deficit. These findings indicate that, despite the observed tendency, female hormone therapy does not significantly alter the risk of these events in the observed female patients.

## Introduction

Cerebral cavernous malformations (CCMs) are vascular lesions of the central nervous system with a prevalence ranging from 0.1% to 0.8% in the general population, making them the second most common after developmental venous anomalies (DVA). CCM may remain asymptomatic (11%–44%) or present with seizures or new focal neurological deficits (FND), with or without associated intracranial hemorrhage (ICH).

Recent studies have led to a paradigm shift in the comprehension of the pathophysiology behind CCM hemorrhages (1). Emerging evidence suggests that the development of ICH or FND in patients with CCM may deviate from conventional understanding. It is likely that these events are initiated by thrombosis within the dilated sinusoidal vessels of CCM with reduced blood flow or an associated venous anomaly (2–4). Recent studies have shown that the use of antithrombotic therapy does not increase the risk of ICH and FND but, on the contrary, may have some benefit (5, 6).

Female hormone therapy encompasses birth control treatment in patients of reproductive age and hormone replacement therapy in postmenopausal patients. It is associated with an increased risk of venous thromboembolic events (VTE) and strokes, attributed to its modulation of the procoagulant, anticoagulant, and fibrinolytic pathways (7, 8) and therefore is often withheld from patients with an increased risk of thrombosis.

However, literature on the impact of female hormone therapy on the risk of ICH or FND in patients with CCM is very limited. Recently, a landmark paper was published by Zuurbier et al. (9) which showed that female hormone therapy is associated with a higher risk of intracranial hemorrhage in patients with CCM, raising the question about the safety of female hormone therapy in patients with CCM. Further studies are therefore urgently needed to guide physicians and patients with CCM and to establish treatment recommendations regarding the use of female hormone therapy in this patient group.

Therefore, the objective of this study was to examine the impact of female hormone therapy on the risk of ICH and FND in patients with CCM in a large prospective database of a tertiary reference center.

## Materials and methods

### Study design

Patients fulfilling the following criteria were eligible and included: (1) radiological and/or histological diagnosis of CCM, (2) available clinical baseline characteristics, (3) medication history, and (4) follow-up data with available magnetic resonance imaging and consultation with a board-certified neurosurgeon or neurologist. Radiological and histological diagnoses of CMs were confirmed either by a board-certified neuro- radiologist and/or neuropathologist.

### Data collection

Patient records were reviewed to assess baseline characteristics: date and age at initial diagnosis, sex, date of surgery for CCM, medical history, cardiovascular and non-cardiovascular comorbidities, and date of last follow-up.

Magnetic resonance imaging (MRI) was examined to determine the location of the CCM, which includes supratentorial, brainstem, cerebellum, and spinal cord locations, as well as the presence of multiple CCMs (mCCM). A diagnosis of familial cerebral cavernous malformation (FCCM) was established in patients meeting one or both of the following criteria: (1) having multiple CCMs or one CCM along with at least one other family member with one or more CCMs; and/or possessing a pathogenic variant in KRIT1, CCM2, or PDCD19 (10).

The cardiovascular comorbidities were differentiated in (1) hypertension, (2) coronary heart disease (CHD) / myocardial ischemia (MI), (3) transient ischemic attack (TIA) / stroke, (4) atrial fibrillation (AF) and other cardiac arrythmias, and (5) artificial heart valves.

Medical history from initial medical records was gathered and treatment and outcomes were tracked through yearly prospective monitoring of hospital records and any available primary care practitioner records.

Retrospectively data for female hormone therapy use was collected from these data sources. In this study female hormone therapy is used as an overarching term for the intake of either birth control treatment in reproductive age [birth control pill, hormonal intrauterine device (IUD), and other hormonal contraceptives] or hormone replacement therapy in postmenopausal women. As birth control pills we included combined estrogen contraception and progesterone only pills. Female hormone therapy use was determined as the prescription and receipt of it at any point after the starting point, but before the first outcome event or the end of follow-up (if an outcome event did not occur).

The starting point was defined as the time of the initial clinical presentation. In the final analysis, we included every female individual with a first-in- a-lifetime diagnosis of CCM which fulfilled the abovementioned inclusion criteria and categorized the type of the first clinical presentation by the symptoms that led to the initial diagnosis into (1) incidental finding, (2) focal neurological deficits, or (3) seizures (regardless of earlier events that might, in retrospect, have been attributable to a CM). We noted as well whether hemorrhage did or did not occur at the initial diagnosis. All results are adjusted for age, brainstem location, initial presentation with hemorrhage and brainstem location.

The primary outcome was defined by a new hemorrhage (confirmed by acute/subacute hemorrhage on brain imaging consistent with the time of symptom onset) or a new focal neurological deficit attributable to the location of the CM (but without evidence of a new hemorrhage on brain imaging). Focal neurological deficits without evidence of a new hemorrhage on brain imaging were included as primary outcome since they might be due to an undetected hemorrhage on imaging or triggered by thrombosis and, thus, might be affected by medical treatment (6, 9). The type of new focal neurological deficit during follow-up was further categorized into (1) sensory deficit, (2) motoric deficit, (3) seizures, (4) cranial nerve deficit, and (5) headache.

Dates and numbers of outcome events (new ICH or FND) were recorded during the follow-up period. Three researchers, identified as SW, SN, and VitS, evaluated these outcome events based on the clinical, radiological, and pathological data available, while remaining unaware of the patients’ use of female hormone therapy. Patients who had undergone surgery were considered until the day of their CCM removal, and those who had lost follow-up were considered up to their last recorded follow-up day. Additionally, a review of the electronic patient chart was conducted to investigate the causes and instances of death.

### Statistical analysis

All statistical analyses were performed using R 4.3.2 (R Core Team, 2023). Descriptive statistics are presented as absolute numbers (*n*) and proportions (%) for categorical variables, whereas continuous variables are shown as mean and standard deviation (SD). The primary endpoint was defined by a new hemorrhage or a new focal neurological deficit (ascribable to the CM location). Time-to-event probabilities were plotted using the Kaplan–Meier method (11). For multivariable survival analysis, the Cox proportional hazards regression model was used (12). The strength of association between the number of outcome events (ICH, FND) and patient age, female hormone therapy, the location of the CCM and mCCM was assessed using a multivariable Poisson regression model with an offset for length of follow-up. A two-tailed *p* value of ≤ 0.05 was considered statistically significant.

## Results

### Baseline characteristics

Three hundred twenty-eight female patients diagnosed with CCM met the criteria and were included in the final analysis. The median age at first presentation leading to a CCM diagnosis was 45.3 years (SD 18.1) (Table 1). CCMs were located in the supratentorial compartment in 227 patients (69.2%), the brainstem in 85 patients (25.9%), the cerebellum in 49 patients (14.9%), and the spinal cord in 19 patients (5.8%). Additionally, 56 female patients (17.1%) had CCMs in multiple anatomical compartments.

**Table 1:** Patients’ baseline characteristics stratified by the use of female hormone therapy.

	Overall	No female hormone therapy (nFHT)	Female hormone therapy (FHT)	*p*-value
*n*	328	272	56	
Age initial diagnosis [mean (SD)]	45.3 (18.06)	46.9 (18.0)	37.8 (16.6)	0.001
Location				
Supratentorial (%)	227 (69.2)	191 (70.2)	36 (64.3)	0.473
Cerebellum (%)	49 (14.9)	39 (14.3)	10 (17.9)	0.641
Brainstem (%)	85 (25.9)	71 (26.1)	14 (25.0)	0.997
Spinal (%)	19 (5.8)	15 (5.5)	4 (7.1)	0.872
Multiple (%)	56 (17.1)	47 (17.3)	9 (16.1)	0.981
Surgery (%)	126 (38.4)	100 (36.8)	26 (46.4)	0.229
Symptoms at initial diagnosis (%)				0.459
Focal neurological deficit	151 (46.0)	124 (45.6)	27 (48.2)	
Epileptic seizure	52 (15.9)	40 (14.7)	12 (21.4)	
Incidental Finding	125 (38.1)	108 (39.7)	17 (30.4)	
Hemorrhage at initial diagnosis (%)	181 (55.2)	148 (54.4)	33 (58.9)	0.637
Focal neurological deficit at initial diagnosis (%)				
Epileptic seizure (%)	18 (5.5)	17 (6.2)	1 (1.8)	0.311
Sensory deficit (%)	41 (12.5)	32 (11.8)	9 (16.1)	0.506
Motor deficit (%)	17 (5.2)	17 (6.2)	0 (0.0)	0.112
Cranial nerve impairment (%)	12 (3.7)	9 (3.3)	3 (5.4)	0.724
Headache (%)	33 (10.1)	23 (8.5)	10 (17.9)	0.059
Comorbidities				
Hypertension (%)	65 (19.8)	56 (20.6)	9 (16.1)	0.556
Coronary heart disease (CHD) / myocardial ischemia (MI) (%)	7 (2.1)	7 (2.6)	0 (0.0)	0.480
Transient ischemic attack (TIA) / stroke (%)	19 (5.8)	17 (6.2)	2 (3.6)	0.640
Atrial fibrillation (AF) and other cardiac arrythmias (%)	7 (2.1)	7 (2.6)	0 (0.0)	0.480
Artificial heart valves (%)	1 (0.3)	1 (0.4)	0 (0.0)	1.000
Other comorbidities (%)				0.734
None	250 (76.2)	207 (76.1)	43 (76.8)	
Hematological disease (%)	14 (4.3)	10 (3.7)	4 (7.1)	
Hematological disease and vascular disease (%)	1 (0.3)	1 (0.4)	0 (0.0)	
Hematological and tumor disease (%)	1 (0.3)	1 (0.4)	0 (0.0)	
Hematological disease and familiar cavernomatose (%)	14 (4.3)	13 (4.8)	1 (1.8)	
Tumor disease (%)	38 (11.6)	32 (11.8)	6 (10.7)	
Familiar cavernomatose (%)	8 (2.4)	7 (2.6)	1 (1.8)	
Drug-taking (%)	2 (0.6)	1 (0.4)	1 (1.8)	

In 125 patients (38.1%), CCM was detected incidentally, while 151 patients (46.0%) presented with focal neurological deficits (FND), and 52 patients (15.9%) presented with seizures. Among the 328 patients, surgical resection of at least one CCM was performed in 126 patients (38.4%). Additional details on the cardiovascular and non-cardiovascular comorbidities of our patient cohort can be found in Table 1.

Among the 328 patients, 56 (17.1%) were receiving female hormone therapy. This included 37 patients (11.3%) on birth control treatments and 19 patients (5.8%) on hormone replacement therapy. Birth control treatments comprised the use of birth control pills in 28 cases (8.5%), hormonal intrauterine devices (IUDs) in 5 cases (1.5%), and other hormonal contraceptives (transdermal estrogen patch and subdermal contraceptive implant) in 3 cases (0.9%) (see Table 2).

**Table 2:** Detailed information on the female hormone therapy treatment in our patient cohort.

	Overall
*n*	328
Female hormone therapy (%)	56 (17.1)
Birth control treatment (%)	37 (11.3)
Birth control pill	28 (8.5)
Hormonal intrauterine device (IUD)	5 (1.5)
Other hormonal contraceptives	3 (0.9)
Hormone replacement therapy (%)	19 (5.8)
None	272 (82.9)

Patients on female hormone therapy were younger (37.8 vs. 46.9 years, *p* < 0.001) and had a longer follow-up time compared to the control group (2025.6 vs. 1400.7 days, *p* < 0.03). The likelihood of ending follow-up due to surgery was higher in patients on female hormone therapy than in the control group (35.7% vs. 30.9%).

There were no significant differences in the number of CCMs, symptoms at initial diagnosis, or comorbidities between the female hormone therapy group and the control group (Table 1).

### Similiar amount of ICH and FND in patients with CCM on female hormone therapy during follow-up

A comparable number of ICH and FND events were observed in patients with CCM who were on female hormone therapy compared with those who were not (see Table 3).

**Table 3:** Outcome stratified by the use of female hormone therapy (FHT) and no female hormone therapy (nFHT).

	Overall	nFHT	FHT	*p*-value
Follow-up length in days [mean (SD)]	1507.5 (1959.6)	1400.7 (1757.7)	2025.6 (2697.6)	0.030
Time-to-primary-event				
First hemorrhage after initial diagnosis in days [mean (SD)]	1241.7 (1788.5)	1161.4 (1650.8)	1631.5 (2324.6)	0.35
First FND after initial diagnosis in days [mean (SD)]	1275.2 (1704.7)	1208.1 (1649.5)	1601.0 (1934.1)	0.86
Hemorrhage in follow-up (%)	80 (24.4)	67 (24.6)	13 (23.2)	0.96
Number of hemorrhages in follow-up [mean (SD)]	0.39 (0.86)	0.38 (0.80)	0.43 (1.11)	0.10
Focal neurological deficit in follow-up (%)	64 (19.5)	51 (18.8)	13 (23.2)	0.560
Number of focal neurological deficits in follow-up [mean (SD)]	0.28 (0.70)	0.26 (0.66)	0.36 (0.84)	0.58
End of follow-up (%)				0.199
Last contact	210 (64.0)	174 (64.0)	36 (64.3)	
Surgery	104 (31.7)	84 (30.9)	20 (35.7)	
Death	14 (4.3)	14 (5.1)	0 (0.0)	
End of follow-up due to death or surgery (%)	118 (36.0)	98 (36.0)	20 (35.7)	1.000

Primary event defined as first intracanial hemorrhage or focal neurological deficit after initial diagnosis. Further outcomes include number of ICH/FND during the follow-up period and end of follow-up analysis in both groups.

A cohort of 328 female patients was monitored from the initial diagnosis of CCM to assess the primary outcomes of ICH or FND associated with CCM. Over the follow-up period of 2025.6 days (FHT) and 1400.7 days (nFHT), there was no significant difference in the number of ICH between patients receiving female hormone therapy (FHT) and those in the control group (nFHT) (mean 0.43; SD 1.11 vs. 0.38; SD 0.8, *p* = 0.1). Similarly, the number of FND was comparable between the two groups, with no significant difference (FHT vs. nFHT: mean 0.36; SD 0.84 vs. 0.28; SD 0.66, *p* = 0.58, see Table 3).

### Similar risk of experiencing a first ICH or, FND in patients with CCM on female hormone therapy

The likelihood of experiencing a first ICH or FND was comparable for both groups of CCM patients, regardless of whether they were on female hormone therapy or not.

**Figure 1:**
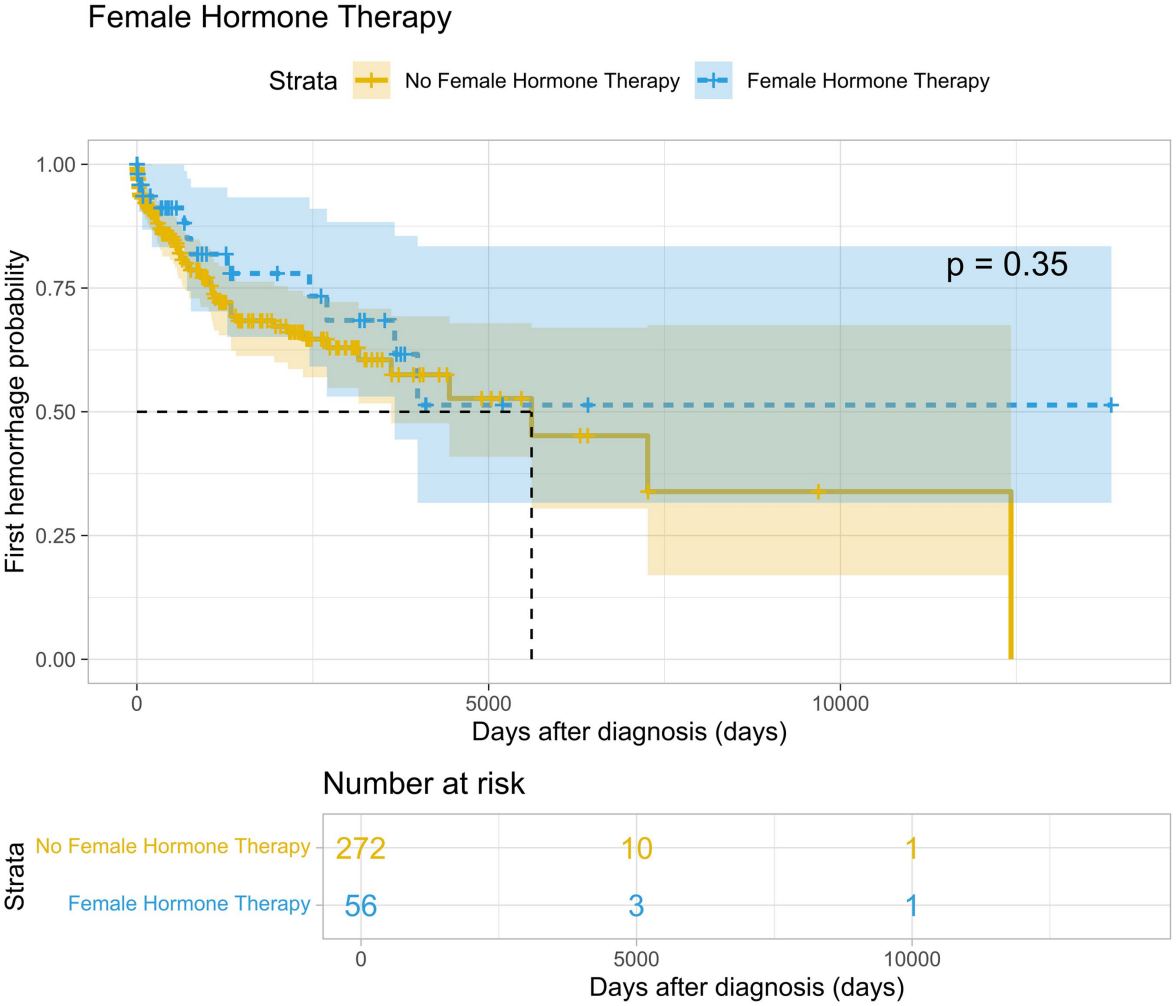
Time-to-event probability for experiencing first hemorrhage. Kaplan–Meier curve visualizing the probability of a first hemorrhage over time in patients receiving female hormone therapy (blue) versus those not receiving hormone therapy (yellow).

**Figure 2:**
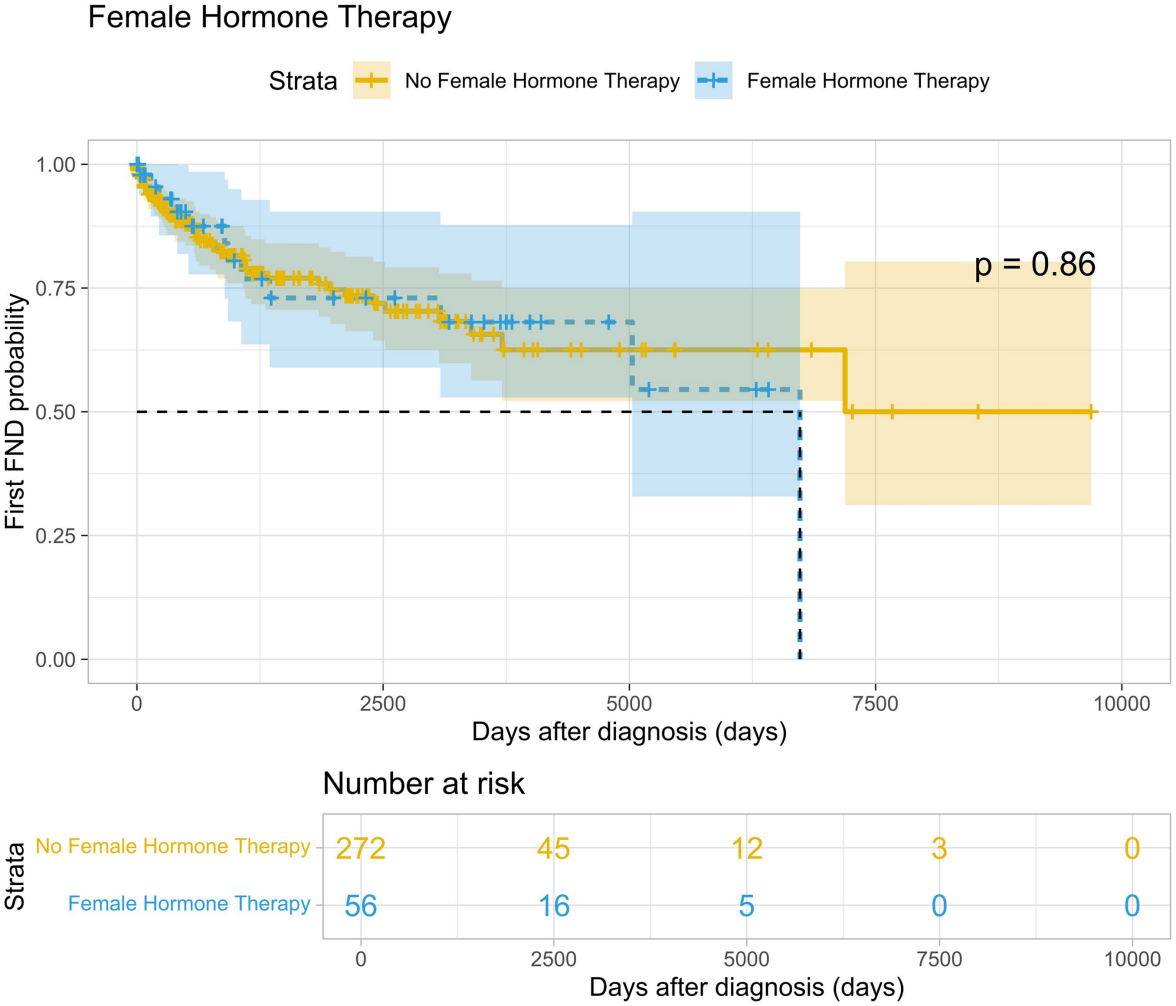
Time-to-event probability for experiencing first focal neurological deficit. Kaplan–Meier curve visualizing the probability of a first focal neurological deficit over time in patients on female hormone therapy (blue) and no female hormone therapy (yellow).

On average, patients in the female hormone therapy group experienced a hemorrhage after 1631.5 days of follow-up (SD 2324.6), compared to 1161.4 days (SD 1650.8) in the control group (*p* = 0.35) (Figure 1). Regarding the risk of developing a FND in the female hormone therapy group, the average time until the first FND was 1601.0 days (SD 1934.1), while in the control group, it was 1208.1 days (SD 1649.5) (*p* = 0,86) (Figure 2).

While there is a trend indicating that ICH and FND tend to occur later in patients receiving FHT compared to the control group, these differences were not statistically significant. The time to the first hemorrhage (*p* = 0.35) and the time to the first FND (*p* = 0.86) did not show a significant association with the use of FHT.

## Discussion

Despite numerous studies concentrating on the exploration of potential risk factors for ICH, or treatment and follow-up approaches for patients with CCM, significant uncertainty persists in clinical practice concerning the use and safety of medications in these patients. This uncertainty arises from the unclear understanding of the pathogenesis of ICH and FND in CCM (3, 4).

In clinical practice, female hormone therapy is often prescribed unquestioningly for patients harboring CCM, despite the known increased risk of thrombosis due to female hormone therapy. Emerging evidence suggests that the development of ICH and FND in individuals with CCM may diverge from the conventional understanding and is more likely to be initiated by thrombosis within the CCM (2, 13). Recent studies showed that patients with CCM who were on antithrombotic therapy had a decreased risk of ICH and FND (5, 6, 14), whereas patients receiving female hormone therapy showed a increased risk of ICH and FND (REF).

This study suggests that patients receiving female hormone therapy do not experience a significantly higher likelihood of intracranial hemorrhage (ICH) or focal neurological deficit (FND). Both the frequency of these events and the likelihood of occurrence were examined, revealing that the rates are comparable between the hormone therapy group and the control group. This finding holds true for both ICH and FND. Additionally, the analysis using a time-to-event Kaplan–Meier curve indicated similar probabilities of events occurring in both groups.

These findings hold significance for clinical practice, providing added reassurance regarding the safety of female hormone therapy. This is particularly relevant for patients experiencing postmenopausal symptoms or requiring birth control treatment, where the therapy is strongly indicated.

Our study’s strengths encompass: (I) the substantial sample size of 328 patients, marking a large cohort to investigate the effects of female hormone therapy in individuals with CCM, (II) the very long follow-up time of 1507.5 person-days in total, and (III) outcome assessment masked to female hormone therapy use.

The limitations of our study are: (I) the intrinsic constraints of retrospective cohorts and incomplete patient information, including missing information of dosage and application type, (II) the potential of selection bias inherent in the retrospective study design, favoring the use of female hormone therapy (i.e., patients using birth control treatment were younger and thus less likely to have other comorbidities), (III) the generally small proportion of patients receiving female hormone therapy (17.1%) and (IV) the frequent omission of recording the contraceptive method in the patient’s medical record.

## Conclusion

This study offers important understandings for the safety of female hormone therapy for women with CCM. Our findings indicate that female hormone therapy does not increase the risk of intracranial hemorrhage (ICH) or focal neurological deficits (FND) compared to patients not receiving hormone therapy. The similar incidence rates and time-to-event analyses suggest that female hormone therapy can be considered safe for patients requiring treatment for postmenopausal symptoms or contraception.

These results are particularly relevant in clinical practice, as they address longstanding concerns regarding the potential risks associated with female hormone therapy in patients with CCM. However, the study’s limitations, such as its retrospective design and the small number of patients on female hormone therapy, highlight the need for further prospective research to validate these findings. Future research should further investigate the effects of different hormone types, routes of administration, and patient characteristics (e.g., co-existing risk factors such as nicotine consumation). Moreover, we recommend increasing the sample size of patients receiving female hormone therapy by pooling data from multiple research centers to enhance the statistical power and generalizability of the findings.

Overall, our findings contribute to a better understanding of female hormone therapy’s role in managing female patients with CCM, offering reassurance for both patients and clinicians.

## Data Availability

The raw data supporting the conclusions of this article will be made available by the authors, without undue reservation.
